# Immunization with truncated envelope protein of Zika virus induces protective immune response in mice

**DOI:** 10.1038/s41598-017-10595-5

**Published:** 2017-08-30

**Authors:** Jian-Feng Han, Yang Qiu, Jiu-Yang Yu, Hong-Jiang Wang, Yong-Qiang Deng, Xiao-Feng Li, Hui Zhao, Han-Xiao Sun, Cheng-Feng Qin

**Affiliations:** 1 Department of Virology, State Key Laboratory of Pathogen and Biosecurity, Beijing Institute of Microbiology and Epidemiology, Beijing, 100071 China; 2 Ji’nan University, Guangzhou, 510632 China; 3 State Key Laboratory of Virology, College of Life Sciences, Wuhan University, Wuhan, Hubei 430072 China

## Abstract

The global spread of Zika virus (ZIKV) as well as its unexpected link to infant microcephaly have resulted in serious public health concerns. No antiviral drugs against ZIKV is currently available, and vaccine development is of high priority to prepare for potential ZIKV pandemic. In the present study, a truncated E protein with the N-terminal 90% region reserved (E90) from a contemporary ZIKV strain was cloned and expressed in *Escherichia coli*, purified by a Ni-NTA column, and characterized by Western blotting assays. Immunization with recombinant E90 induced robust ZIKV-specific humoral response in adult BALB/c mice. Passive transfer of the antisera from E90-immunized mice conferred full protection against lethal ZIKV challenge in a neonatal mice model. Our results indicate that recombinant ZIKV E90 described here represents as a promising ZIKV subunit vaccine that deserves further clinical development.

## Introduction

Zika virus (ZIKV), family Flaviviridae, genus Flavivirus, which was discovered in the Zika forest in Uganda in 1947, is a newly emerging pathogen related to microcephly since 2015 in South America^1^. The first large outbreak of ZIKV occurred on Yap Island, Federated States of Micronesia, North Pacific, in 2007, with 12,000 people affected by the virus^2^. ZIKV caused a major epidemic in the French Polynesia, South Pacific, in 2013–2014, which affected 28,000 people^3^. Not only hundreds of cases of Guillain-Barré syndrome have sprung up in the epidemic of Zika infection, but also an explosion of microcephaly cases among infants born to infected women in Brazil were recorded. Brazil’s Health Ministry has reported more than 3,530 case of microcephaly, including 46 deaths in January 2016^4^. Currently, no specific treatment or licensed vaccine is available for prevention against this pathogenic virus. There is an urgent need for the development of an effective prophylactic vaccine to prevent ZIKV infection^5^.

ZIKV has an approximately 10 kb positive-sense RNA genome, containing a single ORF flanked by the untranslated regions (UTR) at both ends. The ORF is made up of three structural proteins, the nucleocapsid protein (C), envelope protein (E), and membrane protein (M) and seven non-structural proteins (NS1, NS2A, NS2B, NS3, NS4A, NS4B and NS5). The envelope protein (E) of ZIKV is a receptor binding protein, which is the primary determinant of host range, cell tropism and virulence and a major antigen in eliciting neutralizing antibodies during the immune response^6^. The E protein consists of three domains: a structurally central amino terminal domain (domain I, DI), a dimerization domain (domain II, DII) and a carboxyl terminal Ig-like domain (domain III, DIII)^7, 8^. Functional antibody and epitope mapping studies demonstrated that all three domains are antigenic and recognized by neutralizing antibodies that inhibit viral entry process^7^.

The development of flavivirus vaccine has lasted for decade years for some species such as yellow fever virus, Japanese encephalitis virus, west Nile virus, and dengue virus^9^. Live-attenuated vaccine and subunit vaccine are the most effective forms for most flaviviruses till now. The subunit vaccines are more confirmative on preventive effect and time saved because of the successful examples based on the virus protein^10^. For the ZIKV vaccines, different forms of vaccine candidates had been developed recently such as DNA vaccine, inactivated virus vaccine and recombinant adenovirus vector vaccine^11–13^. Larocca *et al*. reported the immunogenicity and protection profile of recombinant DNA and purified inactivated vaccines in murine model^11^. Recently, Kim *et al*. developed another subunit ZIKV vaccines encoding the extracellular portion of the ZIKV envelope gene (E), while the pups born to dams immunized with this subunit vaccine were partially (50%) protected^13^.

Here, we described the fast development of a recombinant subunit vaccine candidate ZIKV E90 based on the most convenient and economic *Escherichia coli* (*E. coli*) expression system. The antigenicity, immunogenicity, and protection efficacy in mice of recombinant E90 profiled in our study suggest further clinical development as promising vaccine candidate against ZIKV.

## Results

### Expression and purification of the recombinant E90 protein

**Figure 1:**
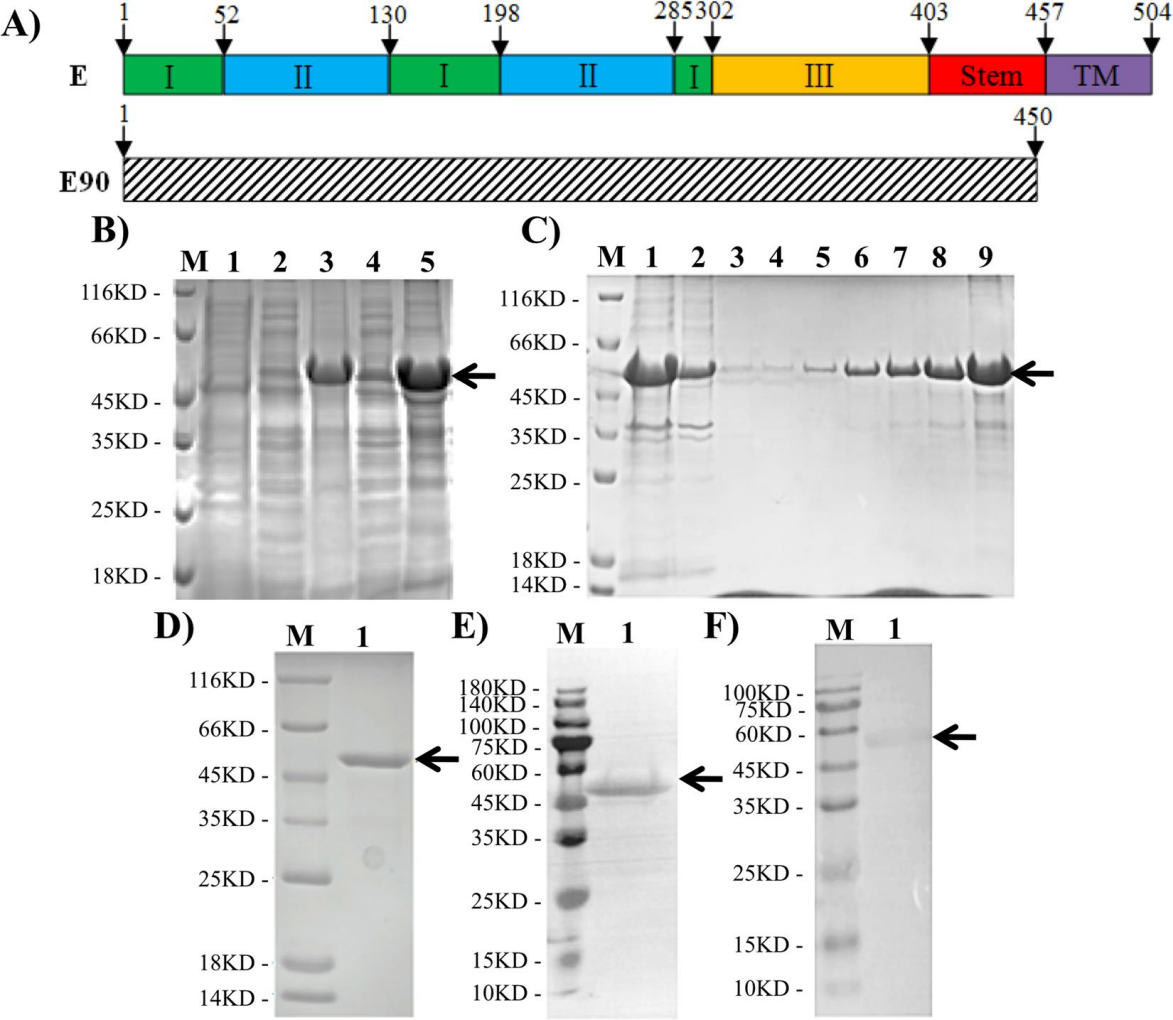
Expression, purification, and characterization of recombinant E90 protein in *E. coli*. (**A**) Schematic diagram of recombinant E90 of ZIKV. The Domains I, II, and III of E protein were shown in green, blue, and yellow, respectively. The stem region and the transmembrane region were in red and purple, respectively. (**B**) SDS-PAGE analysis of the recombinant protein. Lane 1: bacterial lysate before IPTG induction; Lanes 2–3: supernatants and pellets from bacterial lysates at 20 °C; Lanes 4–5: supernatants and pellets from bacterial lysates at 37 °C. The arrow indicated the recombinant E90 protein. (**C**) Purification with a Ni-NTA column. Lane 1: loading samples; Lanes 2: effusion samples; Lanes 3–4: 10 mM Imidazole elution; Lanes 5–6: 50 mM Imidazole elution; Lanes 7–8: 500 mM Imidazole elution. (**D**) SDS-PAGE analysis of the purified ZIKV E90 protein. (**E**,**F**) Western blot analysis of ZIKV E90 using anti-His and anti-flavivirus monoclonal antibodies. M, protein marker. Lane 1, the ZIKV E90 protein.

The recombinant E90 contained the N-terminal 90% of the whole E protein (Fig. 1A), which removed the 3′-end transmembrane domain which probably prevent efficient expression in prokaryotic cells. The corresponding gene fragment was amplified from ZIKV-infected cell cultures and was then cloned into the pET28a expression vector by using standard protocol. *E. coli* BL21 (DE3) was transformed with the engineered plasmid and positive clones were identified by enzyme digestion and PCR-based sequencing. The expression of recombinant E90 protein fused with a His-tag at the N-terminus was induced upon 1 mmol/L of isopropy-b-D-thiogalactoside (IPTG) at 20 °C or 37 °C (Fig. 1B), and the recombinant E90 protein was mainly expressed in inclusion bodies at both temperatures (Fig. 1B). The recombinant protein was approximately 49.3 kDa, which corresponds to the predicted size. Then, the inclusion bodies were solubilized in 8 mol/L urea and the recombinant protein was purified on a Ni-NTA column (Fig. 1C), and the final concentration of purified E90 prepared in our system reached 0.55 mg/ml (Fig. 1D). The further recognized by anti-His tag (Fig. 1E) or anti-flavivirus monoclonal antibody 4G2 (Fig. 1F) by Western blotting assay.

### Immunization of recombinant E90 elicited ZIKV-specific neutralizing antibodies in mice

**Figure 2:**
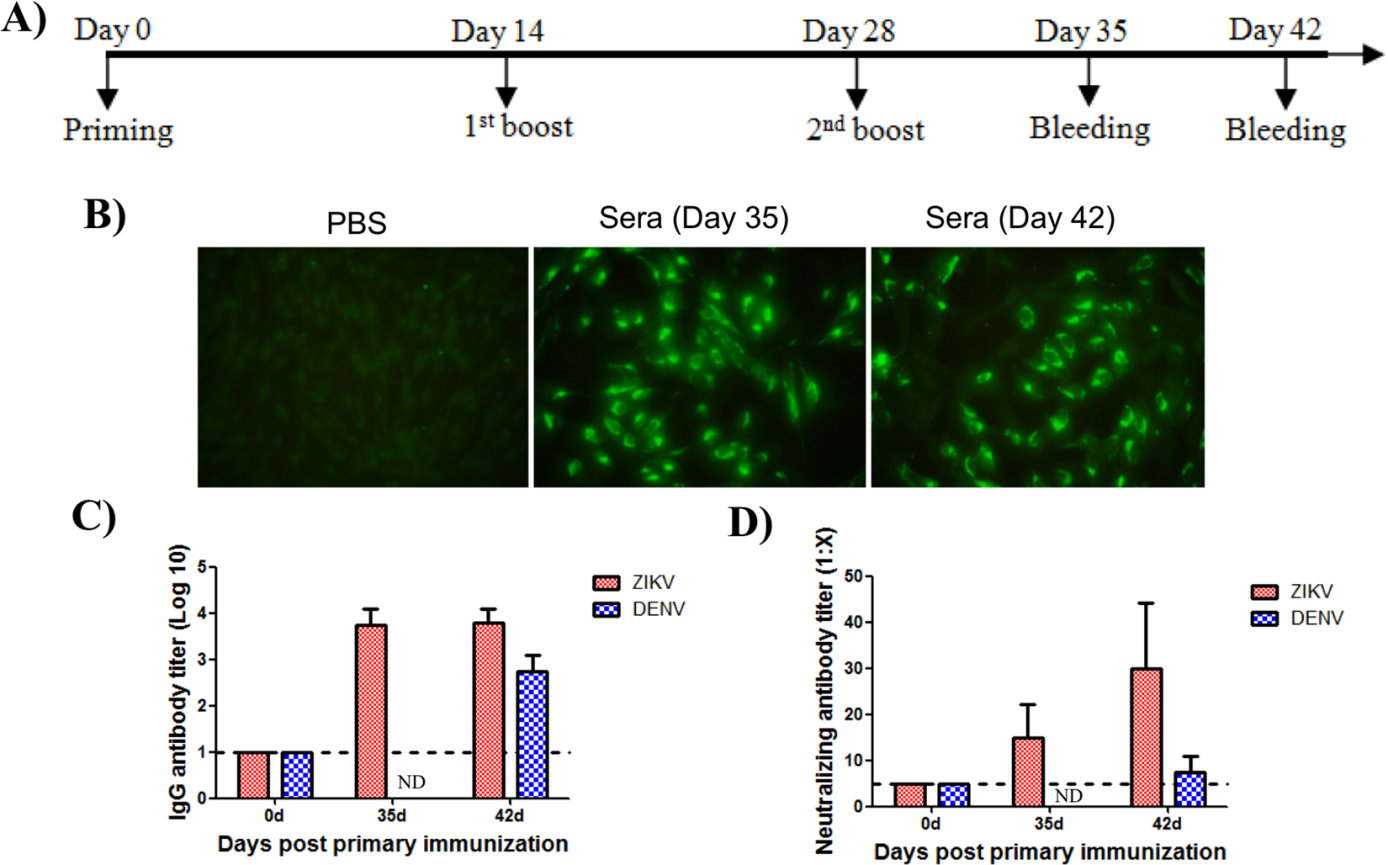
Immunogenicity of recombinant ZIKV E90 in mice. (**A**) Schematic diagram with a timeline of mice experiment. Mice were immunized intraperitoneally (i.p.) with ZIKV E90 with aluminum adjuvant and boosted twice every two weeks. Mice sera were collected 35 and 42 days after the first immunization for antibody assays. (**B**) Indirect immunofluorescence assays were performed by using the sera from the PBS or ZIKV E90-immunized mice. ZIKV-infected Vero cells were incubated with antisera collected at the indicated time points followed by incubation with FITC conjugated goat anti-mouse IgG antibody. (**C**) The IgG antibody titers against ZIKV and DENV were determined by ELISA, respectively. (**D**) The neutralization antibody titers against ZIKV and DENV were determined by a standard plaque reduction neutralization assay, respectively. ND: not determined. Dotted line: the detection limits.

The immunogenicity of the recombinant E90 protein of ZIKV was further assayed in mice that received three doses of immunization according to the specific procedure as shown in Fig. 2A. Indirect Immunofluorescence Assay (IFA) results showed that the sera from mice immunized with E90 could react with viral E protein in ZIKV-infected Vero cells (Fig. 2B). As expected, ZIKV-specific antibodies were not detected from mice immunized with PBS (Fig. 2B). Then, the IgG titers against ZIKV were assayed by standard ELISA, and the results showed that ZIKV E-specific antibodies reached up to 1:10000 at 35 days post immunization, and maintained at least till 42 day post first immunization (Fig. 2C). We also assayed the cross reactivity to DENV, and the antisera from ZIKV E90-immunized mice could also react with DENV with reduced antibody titers (Fig. 2C).

Further, plaque reduction neutralization tests were performed to assay the neutralizing antibody titers in mice immunized with ZIKV E90. As shown in Fig. 2D, the ZIKV-specific neutralization antibodies was detected on 35 days post first immunization and maintained at 42 days after the first immunization. Interestingly, the antisera from ZIKV E90-immunized mice could also neutralize DENV at the tested condition. These results indicated that immunization of ZIKV E90 in mice could induce strong antibody response against ZIKV.

### Immunization of ZIKV E90 confers full protection against ZIKV challenge in neonatal mice

**Figure 3:**
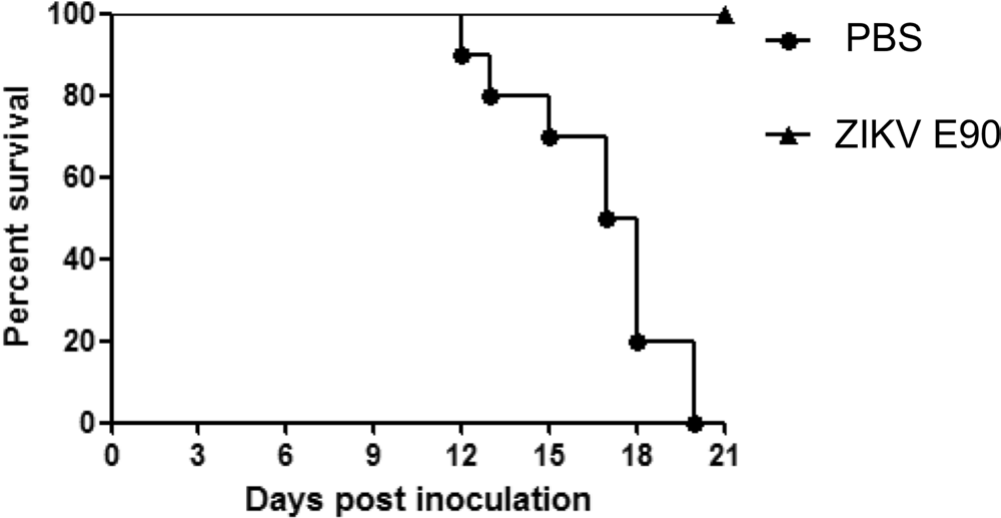
*In vivo* protection against ZIKV challenge in suckling mice model. The antisera from mice immunized with ZIKV E90 or PBS were incubated with an equal volume of ZIKV. Groups of one-day-old BALB/c mice were inoculated intraperitoneally with the mixture described above. Mortality was monitored and recorded daily for 21 days. Kaplan-Meier survival curves were used to display mortality data, and log rank analyses were performed to determine statistical significance between different groups.

Finally, to investigate the *in vivo* protection efficacy of ZIKV E90, a passive transfer experiment was performed using a well established neonatal mouse model as described previously^14^. The antisera from mice immunized with ZIKV E90 or PBS were and mixed with 100 PFU of ZIKV before i.p. administration in 1-day-old neonatal mice. The results showed that the mice administrated with antisera from PBS-immunized mice became sick at day 10 post-inoculation with typical neurological signs, including hind limb paralysis, and died within 20 days post challenge. Mice inoculated with antisera from ZIKV E90-immunized mice had 100% survival rate, none of the infected animals developed any clinical symptoms (Fig. 3). These findings suggested that the ZIKV E90 immunization confers full protection against ZIKV challengeg in the neonatal mouse model.

## Discussions

In this study, we developed and evaluated a truncated version of subunit vaccine of ZIKV, and the promising results warrants further development. Coller *et al*.^15^ demonstrated that the truncated E protein of DENV (DEN-80E) induced protective immune responses in both mouse and non-human primate models. Especially, a proof-of-principle Phase I clinical trial showed that DEN1–80E induced neutralizing antibodies in the majority of subjects with ideal safety proflie^16^. Recently, Kim *et al*.^13^ used codon-optimized E gene as the immunogen and the result from C57BL/6 mice showed that the anti-ZIKV neutralizing titer was up to 1:64. We used an immunocompetent BALB/c neonatal mouse challenge model of ZIKV infection for the *in vivo* protection experiment, which was widely used for DENV and EV71 vaccine evaluation^14^. The importance of E-specific antibodies during protection agaisnt ZIKV has been well demonstrated. Sapparapu *et al*.^17^ recently showed that human monoclonal antibodies isolated from ZIKV patients recognized a panel of unique epitopes on the E protein dimer-dimer interface, and passive transfer of antibodies reduced tissue pathology, prevented placental and fetal infection in mice. Cross reactivity and neutralization between ZIKV and DENV has been well described. For instance, Swanstrom *et al*.^18^ reported human monoclonal antibodies and immune sera derived from dengue patients could neutralize various ZIKV strains, which suggested that ZIKV and DENV share epitopes that are targeted by neutralizing antibodies. Our results showed the antisera from ZIKV E90-immunized mice could also cross reacted and neutralized DENV.

Overall, our preliminary report showed that recombinant ZIKV E90 prepared in *E. coli* expression system could induce protective humoral immune response in mice and protects neonatal mice from wild type ZIKV challenge. In the absence of commercial ZIKV vaccines, this finding may provide a useful strategy in the fast development of a protein-based subunit vaccines against ZIKV^19^. Thinking of antibody-dependent enhancement (ADE) phenomenon existed in both ZIKV and DENV infection, more vaccine candidates need to be rationally designed and evaluated based on current understanding of ZIKV and DENV pathogenesis^20–22^.

## Materials and Methods

### Cells and viruses

Vero and BHK-21 cells were maintained in DMEM containing 10% inactivated fetal calf serum (FCS) plus 100 μg ml/L of penicillin/streptomycin, which were grown at 37 °C in an atmosphere of 5% CO_2_. ZIKV strain SZ01 (GenBank No. KU866423) was isolated from a patient returning from Samoa in 2016^23^. Viral stocks were propagated in Vero cells and titrated in BHK-21 cell by plaque forming assay, and stored as aliquots at −80 °C until use. Experiments with infectious ZIKV were conducted under Biosafety Level 2 facilities at Beijing Institute of Microbiology and Epidemiology.

### Plasmid construction and protein expression

The gene fragment of E90 was amplified by RT-PCR using *in vitro* synthesized genome cDNA of ZIKV strain FSS13025 (GenBank No. JN860885)^24^ as template. The PCR product of 1500 bp was digested with the restriction enzymes *Nde* I and *Xho* I and ligated into the pET28a vector digested with the same restriction enzymes. The resulting construct was confirmed by DNA sequencing. *Escherichia coli* BL21 (DE3) transformed with the plasmid was grown in LB media with shaking at 37 °C. IPTG was added to a final concentration of 1 mmol/L for additional 1–6 h.

### Purification of recombinant ZIKV E90 protein

Inclusion bodies were solubilized in 8 mol/L urea and the proteins were purified by Ni-NTA Agarose. The purity of purified protein was examined by SDS-PAGE and confirmed by Western blot with rabbit anti-His antibody (1:500) or mice anti-flavivirus antibody 4G2^22^ (1:300) as the detection antibodies. The final concentration of the ZIKV E90 protein was determined by using BCA protein assay kit.

### Mice immunization

The mice were obtained from the Laboratory Animal Center, AMMS, China. All of the animal experiments were approved by and performed according to the guidelines of the Animal Experiment Committee of Beijing Microbiology and Epidemiology Institute. A total of 50 μg of recombinant ZIKV E90 protein was mixed with an equal volume of Imject Alum (Thermo, USA). The antigen-adjuvant mixture was administered to 6-week-old female BALB/c mice intraperitoneally and boosted twice at a 14-day interval after the first immunization. The mice sera were collected 7 and 14 days after the last booster and stored at −20 °C.

### Indirect immunofluorescence assay (IFA)

Indirect immunofluorescence assays were performed by using the sera from the PBS or ZIKV E90-immunized mice. Briefly, ZIKV-infected Vero cells were incubated with antisera collected at the indicated time points at a dilution of 1:100 followed by incubation with FITC conjugated goat anti-mouse IgG antibody at a dilution of 1:1000. The cells were then examined under fluorescence microscope.

### ELISA

Quantification of serum ZIKV E-specific IgG antibodies^25^ was performed by indirect ELISA, as described previously using 96-well plates coated with 100 ng of E protein per well^12^. The purified protein E of ZIKV or DENV were used as capture antigens and HRP-conjugated goat-anti-mouse IgG was used as detection antibody respectively. Different dilutions of serum (1:100–1:10000) were used to measure the titre of serum by indirect ELISA.

### Neutralizing antibody assays by PRNT

The serum neutralizing antibody titers were determined by plaque reduction neutralization test (PRNT) in Vero cells cultured in 6-well plates as described before^12^. Serum samples were tested in duplicate at two-fold dilutions in DMEM starting from 1:10 and mixed with an equal volume of 100 PFU/well of the ZIKV or DENV and incubated at 37 °C for 1 h. Determination of PRNT50 end-point titers was based on the serum dilution that led to 50% reduction of the number of plaques compared with the virus control wells.

### *In vivo* protection assay

*In vivo* challenge experiments were performed as previously described^14^. Briefly, the antisera from mice immunized with ZIKV E90 or PBS were incubated with 100 PFU of ZIKV at 37 °C for 1 h. One-day-old BALB/c mice (n = 10 per group) were inoculated intraperitoneally with the virus-sera mixture described above. All mice were monitored daily for clinical symptoms and death for up to 21 days after inoculation.

### Statistical analysis

The antibody titers were log transformed for analysis using GraphPad Prism v5 (GraphPad Software, CA, USA). Kaplan-Meier survival curves were used to display mortality data, and log rank analyses were performed to determine statistical significance between different groups.
